# ADTGP: correcting single-cell antibody sequencing data using Gaussian process regression

**DOI:** 10.1093/bioinformatics/btae660

**Published:** 2024-11-06

**Authors:** Alex C H Liu, Steven M Chan

**Affiliations:** Princess Margaret Cancer Centre, Toronto, Ontario, M5G 1L7, Canada; Department of Medical Biophysics, University of Toronto, Toronto, Ontario, M5G 1L7, Canada; Princess Margaret Cancer Centre, Toronto, Ontario, M5G 1L7, Canada; Department of Medical Biophysics, University of Toronto, Toronto, Ontario, M5G 1L7, Canada

## Abstract

**Summary:**

We present ADTGP, an R package that uses Gaussian process regression to correct droplet-specific technical noise in single-cell protein sequencing data. ADTGP improves the interpretability of the data by modeling the distribution of protein expression, conditioned on equal isotype control counts across cells. ADTGP is written in R and needs only the protein raw counts, isotype control raw counts, and a design matrix to run.

**Availability and implementation:**

ADTGP can be installed from https://github.com/northNomad/ADTGP. It depends on Stan and the R package ‘cmdstanr’.

## 1 Introduction

Antibody-derived tags (ADTs) are antibodies labeled with DNA barcodes that enable the quantification of cell-surface proteins by next-generation sequencing. By staining cells with ADTs before encapsulating single-cells into droplets, it is possible to combine surface protein expression with single-cell transcriptomics (e.g. CITE seq) or genotyping (e.g. Tapestri) data (Stoeckius *et al.* 2017, Li *et al.* 2019).

Typically, ADT panels include isotype (negative) control antibodies. However, the most popular workflows—Seurat and Scanpy for single-cell RNA sequencing, and mosaic for single-cell DNA sequencing—employ central-log normalization (CLR) across antibodies, which disregards the information provided by the isotype controls (Wolf *et al.* 2018, Hao *et al.* 2021, MissionBio 2021b). While other normalization methods that utilize the information of isotype control exist, they usually require log-transformation of the protein sequencing count data (Li *et al.* 2020, Mule *et al.* 2022). Despite its prevalence, log-normalization presents a key issue: as log0 is undefined, a pseudocount (normally *n* + 1) must be added. This adjustment introduces technical errors in cells with varying sequencing coverage and for lowly expressed proteins, as log(*n* + 1) approximates log(*n*) only when *n* is large (Lun 2018, Booeshaghi and Pachter 2021).

To address these issues, we developed ADTGP, an R package that corrects single-cell antibody sequencing data by learning the technical noise through Gaussian process regression of isotype control counts. Unlike other methods that employ log-normalization, ADTGP directly models the raw count data to calculate the posterior distribution of protein expression, conditioned on the isotype control counts being equal across cells. Intuitively, this distribution can be interpreted as the expected protein expression level when all the cells have the same isotype control noise.

## 2 Methods

### 2.1 Model and priors

ADTGP directly models the counts using a negative binomial likelihood with mean μ and overdispersion φ. The negative binomial distribution is widely used in the analysis of biological count data, including bulk RNA sequencing (Robinson *et al.* 2010, Love *et al.* 2014) and single-cell RNA sequencing (Hafemeister and Satija 2019), because of its ability to capture the additional variance commonly observed in biological datasets.

Protein Count ∼ Neg. Binomial(μ, φ)

**Figure 1.**
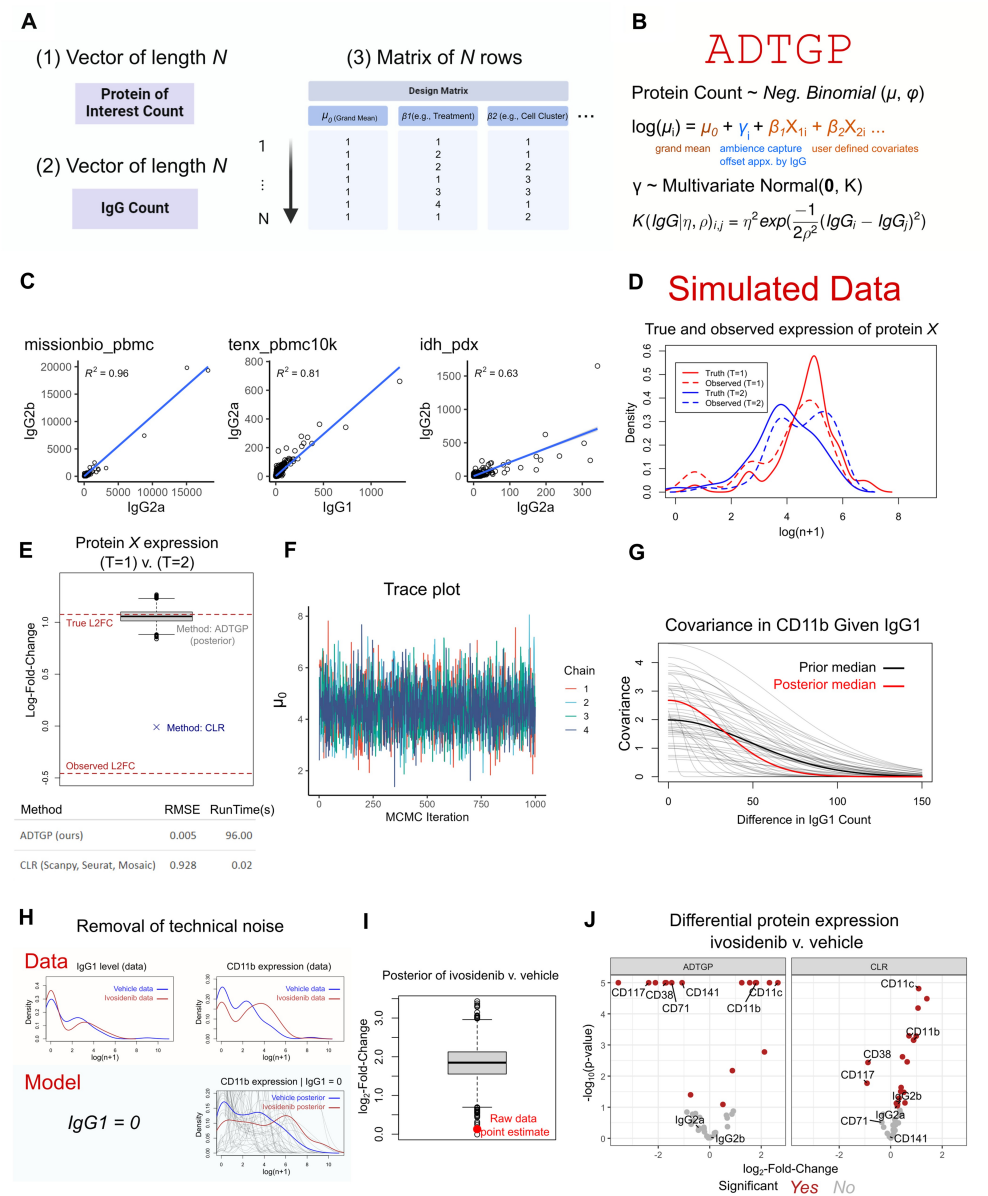
(A) Schematic showing the necessary inputs to run ADTGP. (B) Description of the Gaussian process regression model underlying ADTGP. (C) Dot plots showing the association between isotype controls across the indicated datasets. The linear regression model is performed using the geom_smooth(method="lm") command. (D) Density plots show the true (solid lines) and observed (dashed lines, used to fit the model) expression of protein X in the simulated dataset. (E) Boxplot showing the posterior distribution of log2-foldchange in protein X expression comparing T=1 versus T=2. The bottom dashed line shows the point estimate (mean) from the noisy data before running ADTGP. The top dashed line shows the ground truth. The point marked with an "x" denotes the result using CLR. The bottom table shows performance metrics of the two methods. (F) Trace plot showing the posterior samples of “mu0” from each of the four chains. (G) Distribution of CD11b covariance between cells. The dark curves display the prior and posterior median, respectively. The thin curves show 20 samples from the joint prior distribution of η² and ρ². (H) The top density plots show IgG1 level and CD11b expression in vehicle- and ivosidenib-treated animals in the raw data. The bottom-right density plot shows the conditional distribution of CD11b expression given equal isotype control count. The curves display the density of posterior samples for vehicle and ivosidenib treatments, with prior distribution samples shown for comparison. (I) Boxplot showing the posterior distribution of log2-fold change in CD11b expression comparing ivosidenib versus vehicle treatment. The point marked with a dot shows the point estimate (mean) from the raw data without running ADTGP. (J) Volcano plots showing differential protein analysis using ADTGP (left) and CLR (right). For ADTGP, the log2-fold change is the posterior mean and the significance level is represented using a Bayesian pseudo p-value. For CLR, significance is derived from Student’s t-test.

It uses a log-link function to estimate the mean μ for each cell *i*. There are at least two terms in the linear component of the model: the grand mean μ_0_ (intercept) and γ which represents the deviation from the expectation. Adding additional categorical covariates is straightforward by modifying the input design matrix (Fig. 1A).

$$\log\left(\mu_{i}\right)=\mu_{0}+\gamma_{i}+\beta_{1}X1_{i}+\beta_{2}X2_{i}\cdot \;\cdot \;\cdot$$

Importantly, Gaussian process regression is used in ADTGP to estimate the offset *γ*, which represents the degree of technical noise. Specifically, we use an exponentiated quadratic kernel to learn the covariance between cells based on their isotype control counts. This allows us to model how technical noise is shared between cells with similar isotype control values. The term *η*^2^ describes the maximum covariance between cells and the term *ρ*^2^ parameterizes the rate of decline in covariance as the difference in isotype control counts increases (Fig. 1B).

$$\begin{array}{l} \gamma \;∼\;\mathrm{Multivariate}\;\mathrm{Normal}\left(0,\;K\right)\\ K{\left(\text{IgG}\;|\;\eta ,\;\rho \right)}_{i,j}=\eta^{2}\;\text{exp}(\frac{-\rho 2}{2}\;{\left({\text{IgG}}_{i}—{\text{IgG}}_{j}\right)}^{2}) \end{array}$$

The function offers sensible and weakly-informative priors that cover a wide range of possible outcomes. The default prior for *μ*_0_ encompasses an average count from 1 to several tens of thousands. Because raw count is used to calculate the isotype control distance between cells, we strongly recommend tuning the parameter *ρ*^2^ with prior predictive simulations. By default:

$$\begin{array}{l} \mu_{0}∼\;\mathrm{Gamma}\left(7,\;2\right)\\ \varphi \;∼\;\mathrm{Gamma}\left(0.5,\;0.5\right)\\ \eta^{2}∼\;\mathrm{Normal}\left(2,\;1\right)\\ \rho^{2}∼\;\mathrm{Uniform}\left(0,\;5000\right) \end{array}$$

It is important to note that ADTGP assumes a Gaussian noise structure, which aligns well with the smooth trends we observe in our isotype control data. The model’s performance may degrade if certain cell populations exhibit sharp changes or heavy-tailed noise distributions.

### 2.2 Model fitting

ADTGP explores the posterior space with Stan’s (version 2.35) implementation of Hamiltonian Monte Carlo (Team S.D. 2024). First, the function writes and compiles a stan model file using the input designed matrix and priors. Second, the function fits the model to the data using Stan’s No-U-Turn Sampler through the R interface (version 4.4) with the package cmdstanr (version 0.7.0) (Johnson 2023, Team, R.C 2024). Third, the model draws posterior samples for every combination of covariates in the model. Finally, the function returns a list of two elements: (i) the CmdStanFit object and (ii) the posterior samples stored in a matrix.

### 2.3 Real world datasets

The package provides raw protein sequencing count data from two publicly available (‘missionbio_pbmc’ and ‘10x_pbmc10k’) and one in-house generated dataset (‘idh_pdx’) (Genomics 2018, MissionBio 2021a). The in-house generated ‘idh_pdx’ dataset is derived from human-enriched cells extracted from the bone marrow of mice transplanted with a mix of *IDH1*-mutated and *IDH2*-mutated human acute myeloid leukemia (AML) cells. These mice were treated for eight weeks with either a vehicle control or ivosidenib, a mutant IDH inhibitor, to induce the differentiation of AML cells. The cells were stained with the TotalSeq-DTM Heme Oncology Cocktail with an additional TotalSeqTM-D0392 anti-human CD15 (SSEA-1) antibody and processed with MissionBio’s Tapestri single-cell DNA + protein sequencing platform. All three datasets can be loaded into the R environment using the data() command.

### 2.4 Simulated dataset

In the simulated example, expression of the protein X is two-fold higher in treatment 1 than in treatment 2; both treatments share the same isotype control noise.

$$\begin{array}{l} X1\;∼\;\mathrm{Neg}.\;\mathrm{Binomial}\left(200,\;1\right)\\ X2\;∼\;\mathrm{Neg}.\;\mathrm{Binomial}\left(100,\;1\right)\\ \text{IgG}1,\;2\;∼\;\mathrm{Neg}.\;\mathrm{Binomial}\left(20,\;5\right) \end{array}$$

To simulate *N* cells with correlated *X* and IgG for each treatment, we first draw *N* quantiles *P* from a *Uniform(0, 1)* distribution, and use *P* to extract samples from simulated *X* and IgG.

$$\begin{array}{l} P1,2\;∼\;\mathrm{Uniform}\left(0,\;1\right)\\ {\left[\text{IgG}1,\;X1\right]}_{i=1\ldots N}=\left[\text{Quantile}\;\text{IgG}1\;\left(P1\right),\;\text{Quantile}\;X1\;\left(P1\right)\right]\\ {\left[\text{IgG}2,\;X2\right]}_{i=1\ldots N}=\left[\text{Quantile}\;\text{IgG}2\;\left(P2\right),\;\text{Quantile}\;X2\;\left(P2\right)\right] \end{array}$$

Next, we simulate a scenario where cells sequenced from treatment 2 tend to have higher isotype control noise than treatment 1, such that it masks their true difference in *X*. To do this, we draw 50 cells from [IgG1, *X*1]_*i*__=1…__*N*_ with the probability-weight of each cell *i* being scaled inversely to IgG1, and draw another 50 cells from [IgG2, *X*2]_*i*__=1…__*N*_ with the probability-weight of each cell *i* being scaled proportionally to IgG2. To reproduce this simulation, use the function ADTGP_RunSimulation().

## 3 Results

### 3.1 IgG counts covary between cells across datasets

One major challenge of single-cell protein sequencing data is the technical noise that masks biological variations. Previously, Mulè and colleagues demonstrated that protein-specific noise originates from the encapsulation of unbound antibodies during droplet generation (Mule *et al.* 2022). Consistent with their observation, we found that read counts for negative control antibodies correlate strongly between cells in both publicly available datasets and our in-house generated data (Fig. 1C). This encapsulation of unbound antibodies in single-cell droplets leads to overestimation of protein expression and inflates the expression variability. Therefore, it would be extremely useful to obtain the distribution of protein expression where all the cells share the same isotype control counts.

### 3.2 ADTGP usage example

Cells with similar isotype control counts share a comparable de-gree of unspecific binding, but to a lesser extent than cells of the same isotype control count. The covariation falls off as any two cells become increasingly dissimilar in their isotype control count. ADTGP uses gaussian process regression to learn the covariance between cells as a function of their isotype control counts.

To illustrate the utility of ADTGP, we simulated two cell populations with different expressions of protein X (solid lines, Fig. 1D). We masked this difference by adjusting the isotype control noise: increasing it in T = 2 and decreasing it in T = 1 (dashed lines, Fig. 1D). From the masked data, it appears that the expression of protein X is higher in T = 2 than T = 1 (Fig. 1E, top), which is opposite from the truth. By learning the covariation between isotype control count and protein X, ADTGP accurately captures the true variation between the two populations while CLR failed to detect their differences (Fig. 1E, top). ADTGP sampled four independent Markov chains in 96 seconds on a standard Windows computer and reduced the root-mean-squared-error by 185-fold compared with CLR (Fig. 1E, bottom).

To test ADTGP in a real-world dataset, we focus on how treatment with ivosidenib, a mutant IDH1 inhibitor, can induce the differentiation of *IDH*-mutated AML cells in vivo. As a straight-forward example, we will analyze the expression of CD11b, a surface protein whose expression is associated with the maturation of myeloid cells. First, we use the command data(‘idh_pdx’) to load the dataset into the R environment. Next, we prepare the design matrix containing two columns: the first column is named “mu0” which represents the intercept (grand mean), and the second column is named “T” for Treatment. The column “T” contains the integers 1 and 2, which represent treatment levels for vehicle and ivosidenib, respectively.

To run the model, we pass the raw CD11b counts, and the isotype control counts (IgG1) as vectors, along with the design matrix we prepared in the previous step into the function ADTGP. The numbers of warmup iterations and samples to draw can be specified using the iter_warmup and iter_sampling arguments in ADTGP (iter_warmup = 3000 and iter_samples = 1000 in this example). The function returns a list containing the elements: (i) CmdStanFit object, and (ii) a matrix of posterior samples for CD11b expression in vehicle and ivosidenib treated animals, conditioned on the same technical noise.

Loading the CmdStanFit object shows that the model converged well, where all Rhat values are equal to 1. We can use the $draws command on the CmdStanFit object to extract posterior samples of any parameter. For example, $draws(“mu0”) returns the posterior samples of the intercept across the four chains. By examining the trace plots, the chains appear to mix well and are healthy (Fig. 1F). The model indicates that if cells differ in their isotype control count by 100, their covariance in CD11b expression approaches zero (Fig. 1G).

Plotting the posterior distribution is convenient because ADTGP returns posterior samples from every combination of covariates in the design matrix (Fig. 1H, bottom). Here, we also overlay samples from the prior distribution, which can be simulated using the function ADTGP_prior. After normalizing the technical noise with ADTGP, wd find that the posterior median log2-fold-change of CD11b expression in the ivosidenib versus vehicle treated animals is 1.85, which is significantly higher than the point estimate of 0.13 from the raw data (Fig. 1I). Altogether, ADTGP can uncover true biological variation and improve interpretability of the data by removing droplet specific isotype control noise (Fig. 1H).

To further evaluate ADTGP's performance, we compared it to CLR by performing differential protein expression analysis on all proteins in the dataset. Both methods identified increased expression of key myeloid differentiation markers, such as CD11b and CD11c, and reduced expression of the immature stem-cell marker CD117 (Fig. 1J). However, CLR incorrectly identified two negative control antibodies (IgG2a and IgG2b) as significantly differentially expressed, casting doubts on its reliability (Fig. 1J).

In contrast, ADTGP not only avoided these false positives but also identified additional differentially expressed proteins, including CD71 and CD141 (Fig. 1J), which CLR missed. This demonstrates ADTGP's ability to reveal masked biological variations while minimizing spurious findings.

## 4 Conclusion

We developed ADTGP, an R package that utilizes Gaussian process regression to model and correct technical noise from raw protein sequencing count data. The output of ADTGP is intuitive to understand and the program is highly convenient to use, requiring only three inputs: the raw protein and isotype control counts, and a design matrix.
